# A systematic review and meta-analysis comparing the diagnostic accuracy of initial RT-PCR and CT scan in suspected COVID-19 patients

**DOI:** 10.1259/bjr.20201039

**Published:** 2021-03-02

**Authors:** Manish Devendra Mair, Mohammed Hussain, Saad Siddiqui, Sudip Das, Andrew Baker, Peter Conboy, Theodoros Valsamakis, Javed Uddin, Peter Rea

**Affiliations:** 1 Department of Otorhinolaryngology, University Hospital of Leicester, Leicester, UK

## Abstract

**Objective:**

To perform a systematic review and meta-analysis to compare the diagnostic accuracy of CT and initial reverse transcriptase polymerase chain reaction (RT-PCR) for detecting COVID-19 infection.

**Methods:**

We searched three databases, PubMed, EMBASE, and EMCARE, to identify studies reporting diagnostic accuracy of both CT and RT-PCR in detecting COVID-19 infection between December 2019 and May 2020. For accurate comparison, only those studies that had patients undergoing both CT and RT-PCR were included. Pooled diagnostic accuracy of both the tests was calculated by using a bivariate random effects model.

**Results:**

Based on inclusion criteria, only 11 studies consisting of 1834 patients were included in the final analysis that reported diagnostic accuracy of both CT and RT-PCR, in the same set of patients. Sensitivity estimates for CT scan ranged from 0.69 to 1.00 and for RT-PCR varied ranging from 0.47 to 1.00. The pooled estimates of sensitivity for CT and RT-PCR were 0.91 [95% CI (0.84–0.97)] and 0.84 [95% CI (0.71–0.94)], respectively. On subgroup analysis, pooled sensitivity of CT and RT-PCR was 0.95 [95% CI (0.88–0.98)] and 0.91 [95% CI (0.80–0.96), *p* = o.ooo1]. The pooled specificity of CT and RT-PCR was 0.31 [95% CI (0.035–0.84)] and 1.00 [95% CI (0.96–1.00)].

**Conclusion:**

CT is more sensitive than RT-PCR in detecting COVID-19 infection, but has a very low specificity.

**Advances in knowledge:**

Since the results of a CT scan are available quickly, it can be used as an adjunctive initial diagnostic test for patients with a history of positive contact or epidemiological history.

## Introduction

An outbreak of unexplained pneumonia started in the city of Wuhan, China, in the month of December 2019. The illness was named COVID-19 by World Health Organization (WHO). Human coronaviruses belong to the order Nidovirales, family Coronaviridae, subfamily Coronavirinae, and either genus *Alphacoronavirus* or *Betacoronavirus*. They typically cause transient respiratory or gastrointestinal illness.^1^ Coronaviruses are transmitted by respiratory aerosols and usually produce mild upper respiratory infections.^2^ On 30th January, 2020, the WHO pronounced COVID-19 as a public health emergency of international concern^3^ and on 12th March, the WHO declared COVID-19 to be a pandemic.^4^ The R0 (the basic reproductive ratio) of COVID-19 varies from 2.2 to 3.9^5^ making the virus highly transmissible and from December 2019 to 30th October 2020, the virus has infected 45 428 731 people across the world.^6^ Unfortunately, at present, there are no antiviral medications or vaccines available against this virus.

In view of this high transmission rate and rising number of infected patients, accurate diagnosis of the disease is of paramount importance. Moreover, early identification and isolation of these patients would reduce the global burden of the disease. Reverse transcriptase polymerase chain reaction (RT-PCR) is considered as the gold standard diagnostic test for COVID-19 infection. However, it is time-consuming and accuracy may vary because of differing laboratory techniques. Nonetheless, the availability of RT-PCR kits is limited and there might be a manufacturing defect, resulting in inaccurate results, as reported in USA.^7^ In the meantime, a Chinese study with more than 1000 patients has shown that Computed Tomography (CT) has high sensitivity for detecting COVID-19 infection and should be used as a screening tool for COVID-19 infection in epidemic areas.^8^ However, the American College of Radiology, Royal College of Radiology, Society of Thoracic Radiology and American Society of Emergency Radiology has recommended against the use of CT as a first-line test to diagnose COVID-19.^9–11^ These guidelines suggest that CT scan has low pick up rate of COVID-19 infection in asymptomatic patients with positive RT-PCR and a 20% false-negative rate in symptomatic patients.

Despite this ongoing debate, there is no systematic review or meta-analysis reporting direct comparison between RT-PCR and CT scan as a diagnostic tool for detecting COVID-19 patients. Therefore, we performed a meta-analysis comparing the diagnostic accuracy of CT and initial RT-PCR including only those studies with patients who underwent both CT and RT-PCR testing for diagnosis.

## Methods and materials

### Search strategy and selection criteria

This systematic review and meta-analysis was performed following a predefined protocol (available from authors upon request) and reported in accordance with the preferred reporting items for systematic reviews and meta-analyses (PRISMA) checklist.^12^ We searched three databases, PubMed, EMBASE, and EMCARE, to identify studies reporting diagnostic accuracy of both CT and RT-PCR in detecting COVID-19 infection between December 2019 and September 2020. We used the following search terms: “COVID-19,” “SARS-CoV-2,” and “coronavirus” in conjunction with “RT-PCR,” “polymerase chain reaction,” “swab,” “CT,” and “computed tomography.” Boolean operators (NOT, AND, OR) were also used in succession. In addition, other possible additional publications were included by screening the references of all the included studies. Only those studies comparing CT and RT-PCR as a diagnostic test for COVID-19 in the same set of patients were included in the metanalysis However, we excluded case reports and those studies reporting diagnostic accuracy of only one diagnostic test (CT or RT-PCR). Two reviewers (MM and MH) independently screened the literature search and assessed each study for inclusion. Any disagreement was resolved by consulting a third investigator (SS). The reference standard for diagnosis of COVID-19 infection was subsequent RT-PCR test in eight studies and clinical/epidemiological/RT-PCR (WHO criteria) result was used as a reference standard in three studies.

### Data extraction and analysis

Two authors (MM and MH) independently extracted data from the included studies. The data noted was first author, publication time, city, sample size, mean or median age of patients, true negative, true positive, false negative, and false positive results of both the diagnostic tests. The extracted data were checked by another author (SS). Any disagreement between two authors was resolved through discussion with the third author.

### Assessment of methodological quality

The methodological quality of each included study was assessed using the QUADAS-2 tool on RevMan v.5.3 (Cochrane collaboration, Copenhagen, Denmark).^13^ Quality assessment was not used to exclude studies from the analysis. Domains like participant selection, index test, reference test, and flow and timing were assessed for risk of bias. Applicability was also assessed for the first three domains, namely participant selection, index text, and reference test. Within each domain, the questions were answered with “yes,” “no,” or “unclear,” and the bias risk for each domain was classified as “low,” “high,” or “unclear.

### Statistics

We performed data analyses using Stata v.12. Two-by-two contingency tables were constructed for all included studies. The true positives, false positives, false negatives, and true negatives of the index test based on the study authors’ pre-specified thresholds were noted. The sensitivity and specificity with 95% CI were calculated for each method in each study. Forest plots were produced to show the variation of sensitivity and specificity estimates together with their 95% CI. A meta-analysis for each index test interpretation was performed separately using a mixed effects logistic regression bivariate model, where the logit transformed sensitivities and specificities were modelled.^14^ The summary sensitivity and specificity (summary operating point) with their 95% CIs were then calculated, along with diagnostic odds ratio (DOR), positive (LR+), and negative (LR-) likelihood. Hierarchical summary Receiver operating curve (HSROC) curves were developed presenting the summary points, 95% confidence regions, and 95% prediction regions. The analyses were undertaken using the “metandi,” commands in Stata (Stata-Corp, College Station, TX, USA).^15^ I^2^ test^16^ was used for assessing the heterogeneity between studies and values of 25%, 50%, and 75% suggested low, moderate, and high heterogeneity, respectively.

### Subgroup analysis

The meta-analysis was repeated including only four studies which had COVID-19 negative patients.

## Results

**Figure 1.**
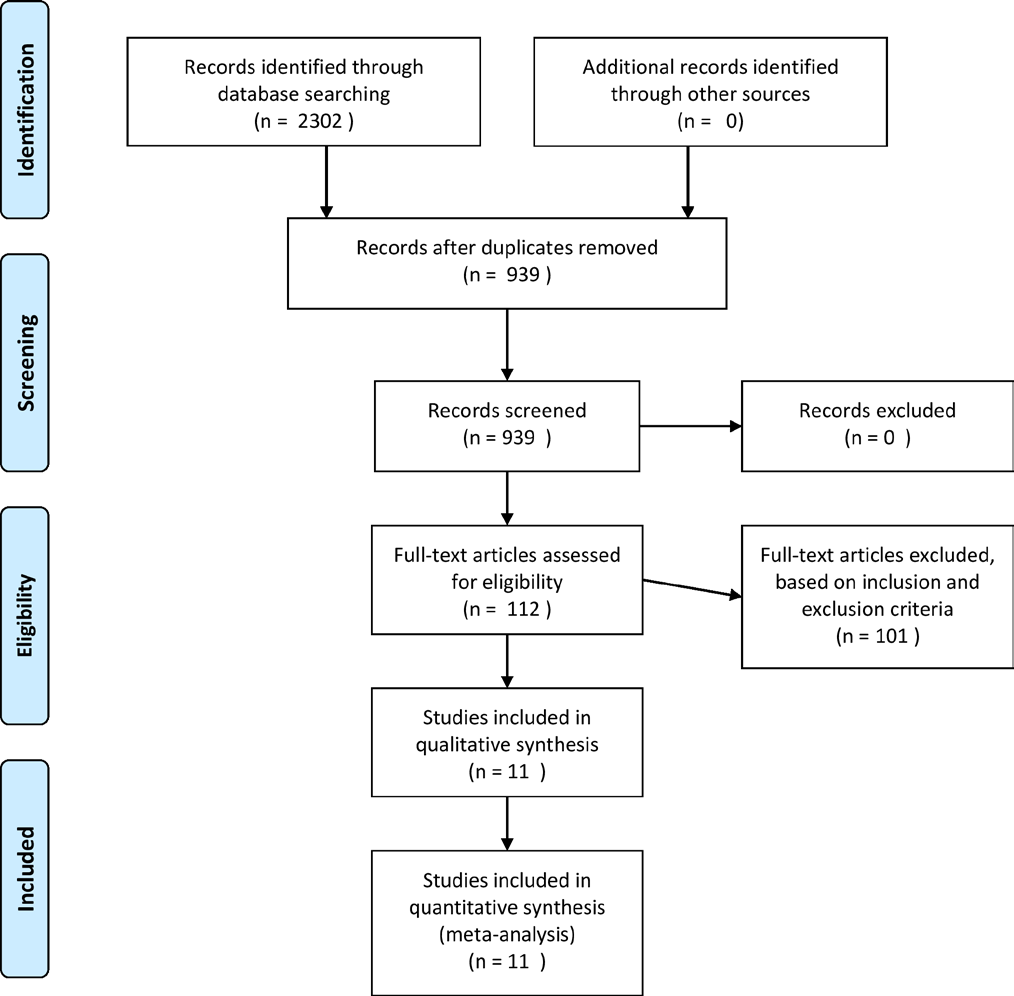
Literature search process is detailed in the PRISMA flow diagram

Results of the search “CT” or computed tomography indentified 2 250 405 results, for “RT-PCR” 1 495 744 reports and from coronavirus 246 906 reports were found. A combination of these terms yielded 2302 papers. After removing duplicate records, 939 articles were included for further assessment. Of these, 112 articles were selected based on the title and abstract and these articles underwent full-text assessment. Seventy-nine studies reported sensitivity of either CT or RT-PCR but not of both, thus were excluded and remaining studies had inadequate data. Based on inclusion criteria, only 11 studies consisting of 1834 patients were included in the final analysis that reported diagnostic accuracy of both CT and RT-PCR, in the same set of patients. The literature search process is detailed in the PRISMA flow diagram (Figure 1). The characteristics of the included studies are presented in Table 1. Overall, the index test was performed in 1834 patients, of which 617 were COVID-19 negative even on subsequent RT-PCR testing. The mean age range for the patients was 33–52 years. Among the 11 studies included in the analysis,^8,17–26^ only four studies^8,17,23,24^ had COVID-19 negative patients and specificity analysis was possible only for the latter. Thus, complete analysis of diagnostic accuracy was done separately as a subgroup analysis including these four studies. In view of this, pooled sensitivity analysis was done twice, once including all the studies and another, including the four studies. The reference standard was taken as initial or subsequent RT-PCR in most of the studies; however, three studies^20,21,26^ considered clinical criteria like epidemiology, symptoms, and ±RT PCR as their reference standard. These studies have taken into consideration the travel history, COVID-19 positive contact, and symptoms. Among these three studies, one study^20^ has ruled out other causes of viral infection like influenza A and B, parainfluenza, respiratory syncytial virus, rhinovirus, adenovirus, and four common coronavirus strains known to cause illness in humans (HKU1, NL63, 229E,and OC43) as well as on swab.

**Table 1.** Demographic characteristics of the studies reporting diagnostic accuracy of computed tomography(CT) and reverse transcriptase-polymerase chain reaction (RT-PCR)

Author	Type of study	Month of publication	Sample size	COVID negative patients	Mean Age	Male	Female	Reference standard taken
He et al^17^	Retrospective	Apr-20	82	48	52/37	49	33	RT-PCR
Fang Y et al^18^	Retrospective	Apr-20	51	0	45	29	22	RT-PCR
Bernheim et al^19^	Retrospective	Feb-20	121	0	45	61	60	RT-PCR
Jian Wu et al^20^	Retrospective	Apr-20	80	0	46.1	39	41	Epidemiology/clinical/RT-PCR
Xie et al^21^	Retrospective	Apr-20	19	0	33	8	11	Clinical
Tao Ai et al^8^	Retrospective	Feb-20	1014	399	51	467	547	RT-PCR
Chan et al^26^	Retrospective	Jan-20	6	0	40.6	3	4	Clinical
Zhang et al^29^	Retrospective	Mar-20	14	0	41	7	7	RT-PCR
Gietema et al^24^	Prospective	Apr-20	193	110	66	113	80	RT-PCR
Long et al^23^	Retrospective	May-20	87	51	44.8	20	16	RT-PCR
Xie et al^25^	Retrospective	Apr-20	167	0	–	–	–	RT-PCR

### Methodological quality of included studies

**Figure 2.**
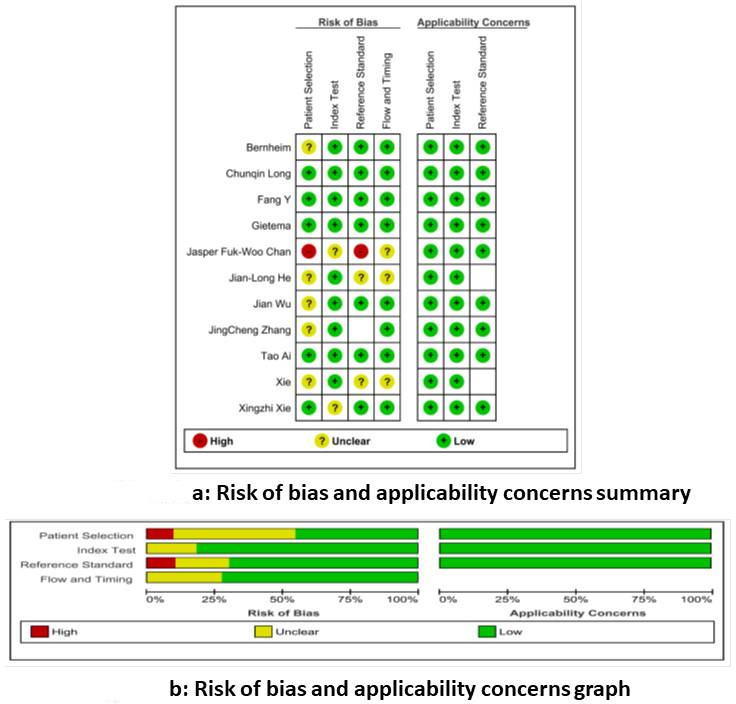
a and b: Risk of bias and applicability concerns summary and graph.

Sources of bias in the accuracy of the index text were mainly related to patient selection. This selection bias might impact the accuracy of both these tests. The risk of bias and applicability concerns summary and graph are shown in Figure 2a and b, respectively. The reference standard was also a source of limitation as three studies used clinical criteria for patient selection. For comparison between CT and RT-PCR, the lag period between the two tests (CT and RT-PCR) was not known in a few studies. However, all patients underwent both the tests and thus a precise comparison between the diagnostic accuracy of these two tests was possible. The study characteristic of the included study is given in Table 1.

### Findings

**Figure 3.**
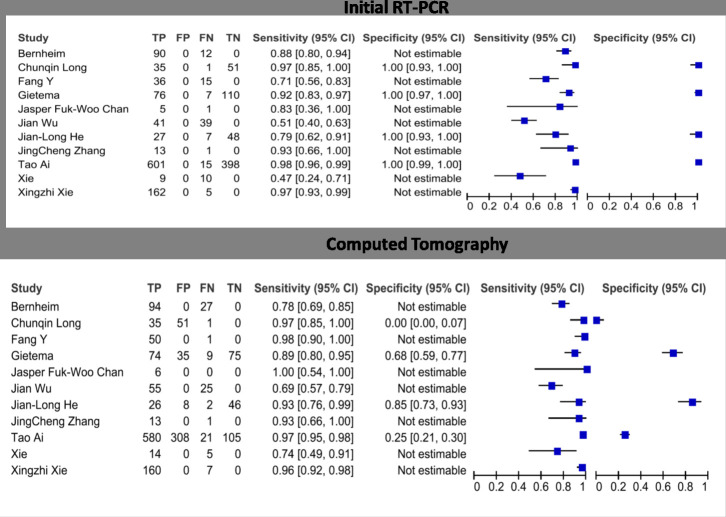
Forest plots of sensitivities and specificities of the CT and RT-PCR for COVID-19 testing.

**Figure 4.**
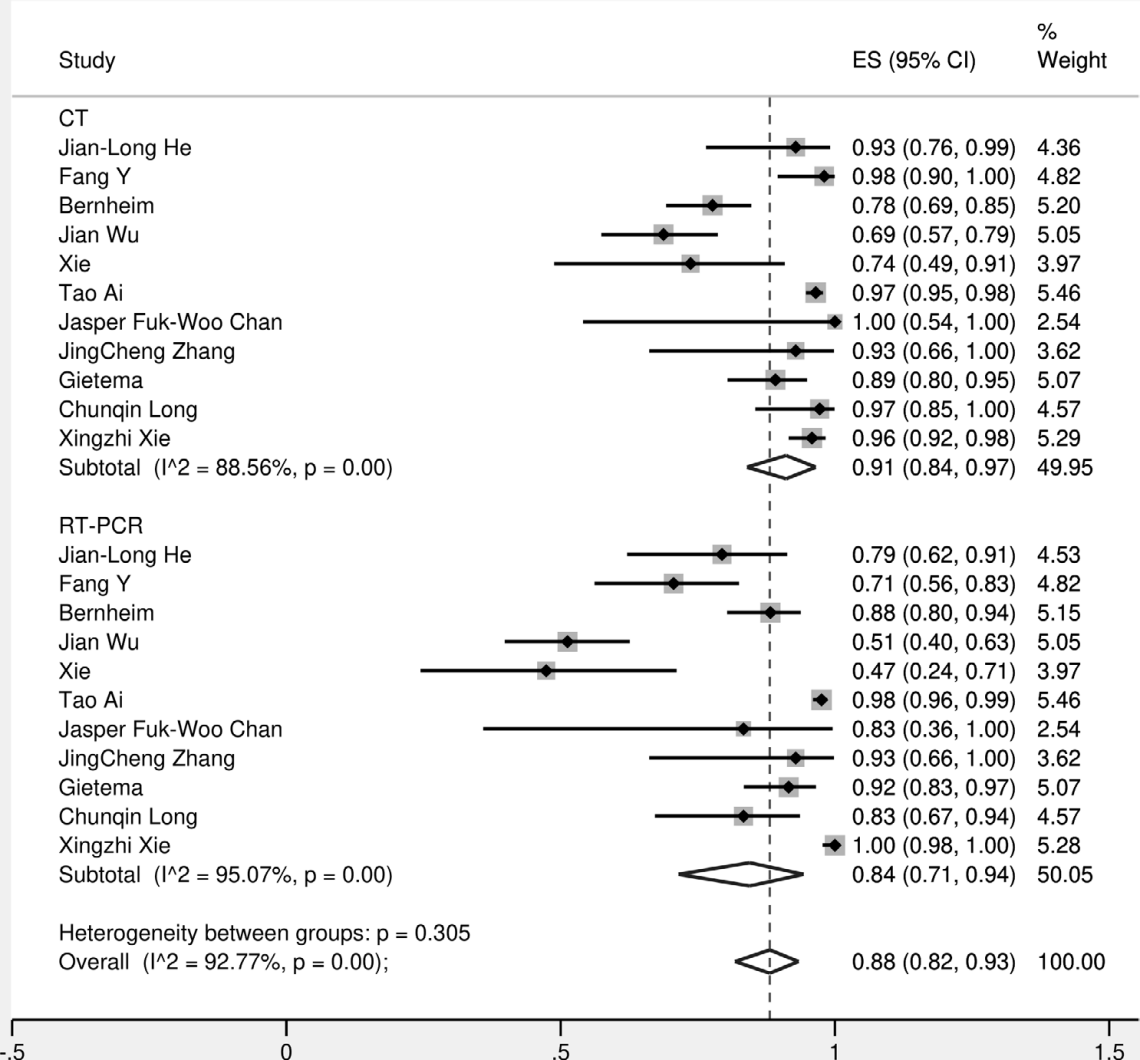
The pooled sensitivity of CT and RT-PCR.

The forest plots of sensitivities and specificities of the CT and RT-PCR for COVID-19 testing are presented in Figure 3. Sensitivity estimates for CT scan ranged from 0.69 to 1.00 and for RT-PCR varied ranging from 0.47 to 1.00. The pooled estimates of sensitivity for CT were 0.91 [95% CI (0.84–0.97)] and RT-PCR was 0.84 [95% CI (0.71–0.94)] (Figure 4).

### Subgroup analysis

**Figure 5.**
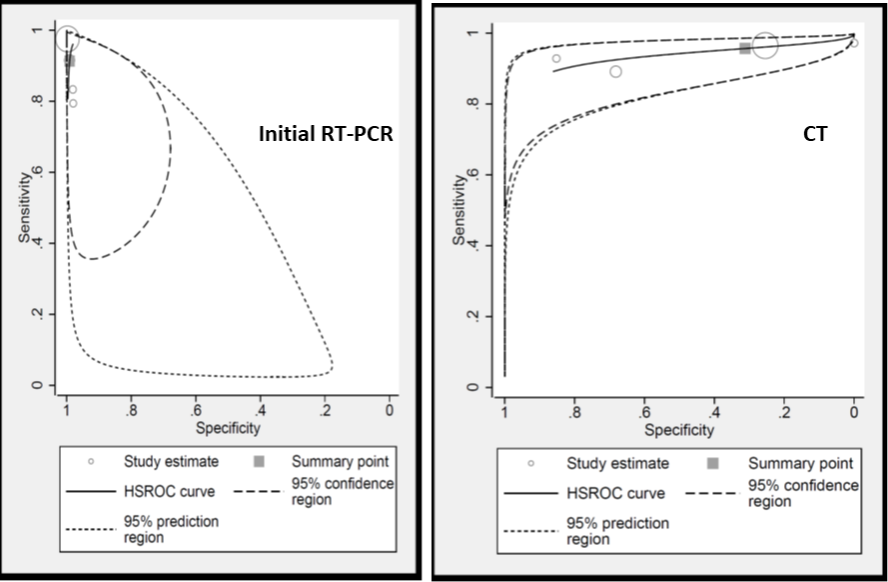
Hierarchical summary Receiver operating curve (HSROC) curves.

This analysis was conducted including only four studies with COVID-19 negative patients. The HSROC curves for each interpretation method are presented in Figure 5a and b.

### Pooled values for CT scan on subgroup analysis

The pooled estimate of sensitivity was 0.95 [95% CI (0.88–0.98)] and specificity was 0.31 [95% CI (0.035–0.84)]. The diagnostic odds ratio was 10.03 [95% CI (1.98–50.68)]. LR +was 1.39 [95% CI (0.66–2.92)] and LR-ve was 0.138 [95% CI (0.05–0.34)].

### Pooled values for RT-PCR on subgroup analysis

The pooled estimate of sensitivity was 0.91 [95% CI (0.80–0.96)] and specificity was 1.00 [95% CI (0.96–1.00)]. The diagnostic odds ratio was 1131.45 [CI (162.79–7863.85)]. LR +was 99.17 [95% CI (27.4–358.95)] and LR-ve was 0.087 [95% CI (0.037–0.207)].

### Comparison of CT vs RT-PCR

It was found that specificity of initial RT-PCR (100%) was higher than CT (31%). However, this is obvious as the reference standard for most of the included studies was subsequent RT-PCR itself. In this way, RT-PCR did not have false-negative cases. With respect to sensitivity, CT (95%) was superior to RT-PCR (91%) with a *p* value of less than 0.05 (Table 2). (HSROC-Figure 5).

**Table 2.** Pooled Sensitivity and Specificity of CT scan and initial RT-PCR

	Sensitivity (95% CI) (11 studies)	Sensitivity (95% CI) (four studies)	Specificity (four studies)	*p* value
CT scan	0.91 (0.84–0.97)	0.95 (0.88–0.98)	0.31 (0.35–0.84)	0.0000
Initial RT-PCR	0.84 (0.71–0.94)	0.91 (0.80–0.96)	1.00 (0.96–1.00)	–

## Discussion

RT-PCR is the gold standard for detection of viral RNA. Nonetheless, the PCR results for SARS‐CoV‐two are related to a number of factors which includes quality of the examination kit, sampling location, sampling volume, transportation, and storage, as well as laboratory test conditions and personnel operation.^27,28^ In addition, as transportation may take some time, there is a delay in the reporting of swab results. Thus, timely isolation of an infected patient may not be possible with RT-PCR alone. Moreover, the person taking the swab is also at risk of infection. The peak of respiratory shedding is seen at the end of first week after acquiring infection, at a similar time to when symptoms develop. However, this shedding is intermittent and thus may lead to an initial negative swab.^29–31^ A Chinese study consisting of more than 1000 patients suggested that there is a 5-day mean delay for an initial negative RT-PCR test to turn positive. In contrast, a CT scan is rapid with immediate reporting. They also suggested that a CT scan is more sensitive than RT-PCR.^6^ Moreover, the risk of transmission to a health care worker might be less with a CT scan as compared to a pharyngeal swab. Therefore, we performed this meta-analysis comparing the diagnostic accuracy of CT scan and initial RT-PCR for detecting COVID-19 infection. In order to have a precise comparison, we have included only those patients who underwent both the tests at initial presentation. .

The hallmark of novel coronavirus pneumonia on a CT scan is the presence of a bilateral subpleural distribution of ground-glass opacities accompanied by interlobular septal thickening.^28,32^ Xie et al^21^ presented chest CT findings of patients with COVID-19 infection who had initial negative RT-PCR results. These patients had ground-glass opacity (five patients) and/or mixed ground-glass opacity and mixed consolidation (two patients). All patients were eventually confirmed to have COVID-19 infection by means of repeated swab tests. Timely isolation was possible in these cases due to their CT scan findings. We found the sensitivity of a CT (95%) scan was significantly higher when compared to initial RT-PCR (91%) on subgroup analysis including four studies(*p*-0.000). Even whilst analyzing all the 11 studies, we found that the sensitivity of RT-PCR (84%) was lower than CT scan (91%), However, CT imaging features are not specific and these features might overlap with other viral pneumonias including influenza, MERS and SARS. On similar lines, the specificity of a CT scan (31%) was lower than RT-PCR (100%) in this meta- analysis. Thus, the CT appearances alone will not obviate the need for viral testing. But it can be used in cases with equivocal results and might help in accessing the severity and recovery of COVID-19 infection. One has to understand the financial burden associated with higher number of CT scans and the radiation exposure to the patients. The radiation exposure can be reduced by using low dose CT scan which is being used for lung cancer screening. However, further research is needed on this topic. Moreover with the seasonal flu caused by influenza and second wave of COVID-19 overlapping in the months of coming winter, diagnosis solely based on CT scan is going to be more challenging. This will further reduce the specificity of CT scan due to high false positive report.

Radiologists may use findings of bronchiectasis and pleural effusion to identify patients with influenza A (H1N1) pneumonia and findings of linear opacification, crazy-paving sign, vascular enlargement, and pleural thickening to identify patents with COVID-19 pneumonia.^33^

A Cochrane systematic review by Salameh et al^34^ consisting of 71 studies that included confirmed cases showed a pooled sensitivity of chest CT was 93.1%. In addition, they also reported 13 studies that included suspected cases, the pooled sensitivity of CT in this case was 86.2% and specificity was 18.1%. Similarly, pooled sensitivity of CT scan was high in our study measuring more than 90% and specificity was low at 31%. A retrospective multicenter study by Schalekamp et al^35^ also showed that chest CT analysis using the COVID-19 reporting and data system (CO-RADS) enables rapid and reliable diagnosis of COVID-19, particularly when symptom duration is greater than 48 h. Depending on the cut-off of CO-RADS score, the sensitivity varies between 71 to 92%. Thus, experience of the radiologist reporting the scans and characteristic features of the scans plays an important role in establishing diagnostic accuracy of CT scan for detecting COVID-19.

The limitations of this meta-analysis include significant heterogeneity among the included studies. The I^2^ statistic for CT scan was 88.56% and for RT-PCR was 95.07%. Moreover, most of the included studies are retrospective. There is also a possibility of publication bias as unpublished data are not included in this meta-analysis. Subset analysis of specificity was done for four studies only. But this is the only meta-analysis in which all the patients underwent both tests [CT and RT-PCR] making precise comparison between these tests possible.

## Conclusion

The sensitivity of CT scanning is significantly higher than RT-PCR for detecting COVID-19 infection, however as CT findings are not specific and may overlap with other viral infection, its specificity is relatively low. Thus, it is unlikely to replace RT-PCR as the gold standard test. Since the results of a CT scan are available quickly, it may be useful as an adjunctive initial diagnostic test for patients with a history of positive contact or epidemiology.
